# Management of Accidental Intravitreal Dexamethasone Injection Into the Lens: A Case Report

**DOI:** 10.7759/cureus.36216

**Published:** 2023-03-16

**Authors:** Emanuele Siotto Pintor, Giuseppe Demarinis, Filippo Tatti, Enrico Peiretti

**Affiliations:** 1 Department of Surgical Sciences, Eye Clinic, University of Cagliari, Cagliari, ITA

**Keywords:** surgical case reports, dexamethasone implant, central retinal vein occlusion (crvo), pars plana lensectomy, cataract extraction

## Abstract

An accidental injection of a dexamethasone implant inside the crystalline lens was observed in the right eye of a 63-year-old woman suffering from a macular edema secondary to a central retinal vein occlusion. A 23-gauge pars plana vitrectomy and lensectomy followed by an intraocular lens implantation were performed to carefully remove the lens and save the whole implant in order to preserve its therapeutics effects. A strict follow-up over the following 3 months revealed an improving of macular edema and no postoperative complications. The injection of a dexamethasone implant into the lens could be effectively and successfully managed with a pars plana vitrectomy and lensectomy.

## Introduction

Retinal vein occlusion (RVO) is the second most common retinal vascular disease after diabetic retinopathy [1]. Dexamethasone implant (DEX-I) is currently approved for the treatment of macular edema following central retinal vein occlusion (CRVO) or branch retinal vein occlusion (BRVO) [2]. DEX-I has a favorable safety and tolerability profile, and cataract progression and ocular hypertonia are the two most common adverse effects [2]. Other treatment-related adverse effects reported are rare and include vitreous and retinal hemorrhage, retinal exudates, endophthalmitis, and retinal detachment [1,2], while lens injury is rare but known [3]. When it happens, the development of an iatrogenic cataract is inevitable, and therefore a cataract surgery becomes mandatory. To the best of our knowledge, this is the first case report where three-port pars plana vitrectomy (PPV) and lensectomy have been performed in order to safely remove the cataract and preserve the DEX-I.

## Case presentation

**Figure 1:**
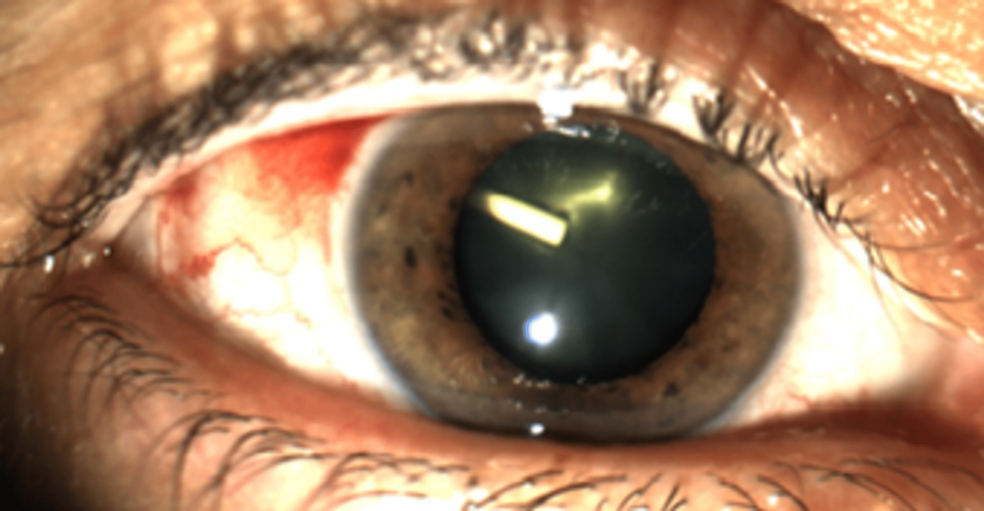
Dexamethasone implant inside the lens. Iatrogenic cataract due to the dexamethasone implant (DEX-I) accidentally injected inside the lens.

A 63-year-old woman presented to our emergency department complaining of severe floaters and decreased vision in her right eye (RE). One week earlier, an intravitreal injection of a dexamethasone implant (DEX -I; Ozurdex®, Allergan Inc., Irvine, CA, USA) was administered due to a macular edema secondary to a non-ischemic CRVO. On presentation, the best corrected visual acuity (BCVA) was count fingers in her RE and 20/20 in the left eye (LE). Intraocular pressure (IOP) was 34 mmHg in the RE and 16mm Hg in the LE. The RE slit-lamp examination revealed an iatrogenic cataract due to DEX-I accidentally injected inside the crystalline lens (Figure 1).

RE fundus examination was not possible due to the opacity of dioptric media; therefore, a decision was made to perform a pars plana vitrectomy lensectomy (PPVL), with intraocular lens (IOL) implantation in the sulcus and reposition of the DEX-I in the vitreous cavity to preserve the therapeutic effect of the implant and avoid another injection (Video 1).

**Video 1:** Pars plana vitrectomy lensectomy surgery video An additional movie file shows PPVL surgery video. DEX-I is still visible in the anterior chamber during the delicate steps of the surgery. Minimally invasive instruments allow cataracts to be removed and DEX-I to be preserved simultaneously. PPVL, with intraocular lens implantation, and reposition of the DEX-I in the vitreous cavity are performed in order to preserve the therapeutic effect of the implant and avoid another injection. DEX-I, dexamethasone implant; PPVL, pars plana vitrectomy lensectomy

**Figure 2:**
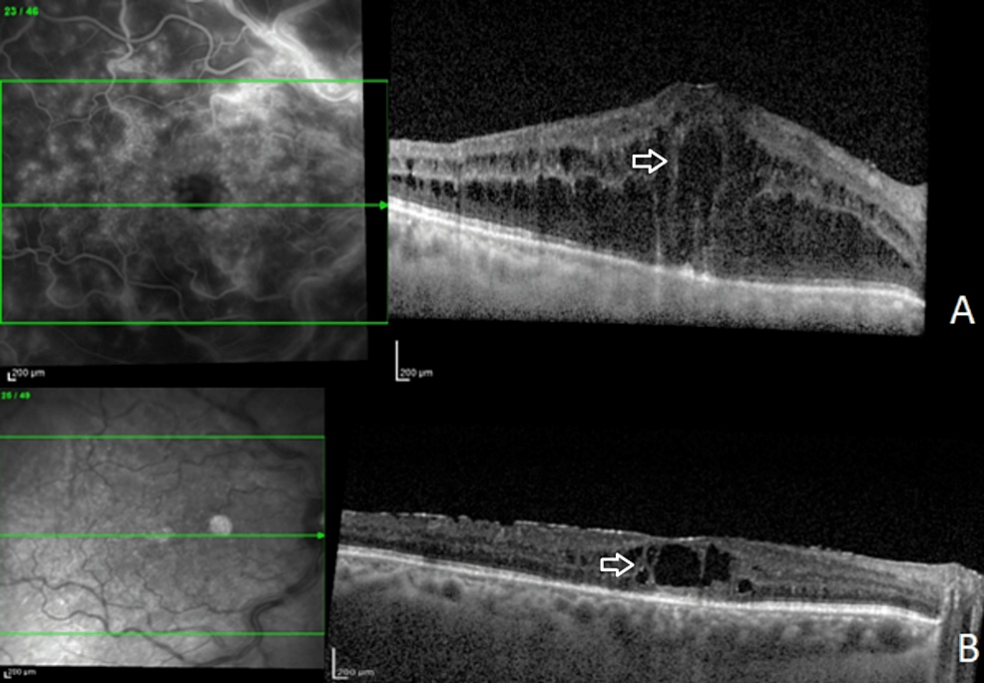
OCT baseline scan (A) The OCT baseline scan shows cystoid macular edema and disorganization of the retinal inner layers. (B) Three months postoperatively, OCT scan demonstrates an IRF reduction. OCT, optical coherence tomography

Under retrobulbar anesthesia, the surgery was performed using a 23G vitrectomy system (Constellation, Alcon Laboratories, Fort Worth, TX, USA). Transconjunctival incisions were created 4 mm from the limbus using trocars in the inferotemporal, superotemporal, and superonasal quadrants. Using the 23G instruments, the anterior vitreous was removed and the lensectomy was performed. The entire DEX-I was preserved and gently moved into the vitreous chamber, and the capsule was preserved for the secondary implantation of IOL. A total vitrectomy was performed, which removed the lens fragments dropped into the vitreous cavity and the residual vitreous. At the end, an IOL was inserted into the ciliary sulcus through a 2.75-mm corneal incision, and an air-fluid exchange was performed. The patient was strictly examined in the postoperative period; on the first postoperative day, the IOL was well-centered and the anterior segment was quiet. On the final postoperative visit (third month), BCVA improved to 20/40, the IOL was still in situ, and the IOP decreased to 14 mmHg. The optical coherence tomography (OCT) scan demonstrated an improvement of the CRVO (Figures 2A, 2B).

## Discussion

DEX-I is a sustained-release implant of 700 mg dexamethasone, which is injected into the vitreous cavity through a 22-gauge (G) needle approximately 3.5 to 4 mm posterior to the limbus [3]. Injuries to the crystalline lens, such as lens touch, iatrogenic cataract formation, lens dislocation, and zonular tears, have been reported as rare complications after intravitreal injections procedure [4]. These can be the consequence of surgeon inexperience, improper technique, or a patient's head movements at the time of the injection [5].

In our opinion, the reported complication resulted from incorrect positioning of the applicator needle during the procedure. This latter, not perpendicular to the scleral surface during injection, advanced into the lens while the dexamethasone implant was being injected. The patient's extremely nervous and anxious state of mind toward the treatment, despite extensive explanations and reassurances, may have played a major role in the event.

A literature review found 22 articles, including case reports and case series, on the accidental injection of the DEX-I into the lens. Based on the eye examination and the state of the insert, two different approaches were chosen: conservative or surgical. As for the former, it depends on several variables such as the state of lens, ocular pressure, and improvement of macular edema. Accelerated cataract development has been observed as the most common complication, followed by ocular hypertension, which was well managed with topical medication. Regarding the benefit of this approach experienced by patients, the literature is discordant, including several case reports that described an initial improvement in visual acuity, despite cataract progression, while other studies considered this approach ineffective [2].

It has been speculated that this is a consequence of the state of DEX-I and its position within the lens. Berarducci et al hypothesized that when DEX-I is not completely localized in the lens, but partly in the vitreous chamber, its release of the drug continued [4]. Coca-Robinot et al. and Sekeroglu et al. similarly observed that, in the same position, DEX-I has a persistent therapeutic effect for up to 6 months [1,5]. In contrast, Baskan et al. and Albuainain et al. observed that DEX-I, placed entirely inside the lens, had no beneficial effect on macular edema [2,6]. In all cases, cataract progression was reported, and consequently a phacoemulsification procedure was performed. In addition, in most of them, either an anterior vitrectomy, following rupture of the posterior capsular bag [3,4], or a vitrectomy for lens material dropped into the vitreous chamber was required [7,8].

On the other hand, several studies have opted to go for phacoemulsification immediately, but with several disadvantages. To begin, the implant repositioning in the vitreous cavity is difficult due to a vitreous resistance, and therefore an anterior vitrectomy is required [3]. Another reason is that the implant may also fragment during repositioning in the vitreous cavity, resulting in impaired drug absorption and increased risk of glaucoma [9]. Lastly, another risk of this approach is the migration of the reinserted implant to the anterior chamber through the posterior capsule defect, resulting in corneal decompensation [10].

In our case, already one week after the injection, a significant cataract combined with a slight increase in eye pressure had been observed. These signs led us to opt for an immediate surgical approach. Therefore, we considered resolving the cataract and preserving the DEX-I at the same time. For this purpose, a PPV and lensectomy technique offered several benefits. Miniaturized instruments (23/25G) allowed a “tailor-made” cataract surgery: the lens material could be safely removed and at the same time it was easier to preserve the entire DEX-I and place it in the vitreous cavity. Moreover, as described by several studies, the possibility of encountering lens fragments in the vitreous chamber during surgery was very high, which is why PPV and lensectomy allowed this complication to be dealt with immediately. Finally, this technique and its timing offered important advantages. By promptly removing the implant from the lens, the iatrogenic chemical corneal endothelium toxicity of DEX-I has been avoided [11]. In addition, lensectomy offered the possibility of removing the cataract without the collateral effect of phacoemulsification, a lower risk of damage to the corneal endothelium and, thanks to an extended vitrectomy, the possibility of removing the vitreous body, an important vascular endothelial growth factor deposit in CRVO patients.

As in previous studies describing the immediate surgical approach, in our case the patient benefited from DEX-I in the following months, and no complications were noticed. Furthermore, compared with a conservative approach, the patient, who underwent cataract surgery immediately, no longer runs the risk of further surgery and thus of a possible Irvine-Gass syndrome.

## Conclusions

In conclusion, this report illustrates the effectiveness of the PPV and lensectomy in case of an accidentally injection of DEX-I inside the lens. The use of minimally invasive vitreoretinal instruments offers the opportunity to accurately remove the cataract and preserve the entire implant. Thanks to this choice, DEX-I can be moved into the vitreous chamber, and the patient can benefit from its therapeutic effect.
